# Association of apolipoprotein E gene polymorphisms with risk of coronary artery disease in a Han Chinese population at middle and high altitude in China

**DOI:** 10.3389/fendo.2026.1765770

**Published:** 2026-02-03

**Authors:** Fanrong Zeng, Xinyi Zhang, Meng Zhang, Hongli Liu, Yuan Li, Fan Ye, Xuejiao Chen, Fangyi Zhu, Lihong Zhai

**Affiliations:** 1 Dongguan Qiaotou Hospital, Dongguan, China; 2 Institute of Neuroscience and Brain Disease, Xiangyang Central Hospital, Affiliated Hospital of Hubei University of Arts and Science, Xiangyang, China; 3 Qinghai Provincial Cardiovascular and Cerebrovascular Disease Specialist Hospital, Xining, China

**Keywords:** *APOE* gene, coronary artery disease (CAD), gene polymorphism, Han Chinese, independent predictor

## Abstract

**Introduction:**

This study investigated the impact of *APOE* gene polymorphisms on the development of coronary artery disease (CAD) in the Han Chinese population at middle and high altitudes, focusing on lipid level regulation and atherosclerosis.

**Methods:**

This retrospective case-control study involved 628 CAD patients and 628 matched controls without CAD. ApoE genotyping was conducted using PCR-chip technology, and genotype and allele frequencies were compared between groups. Multivariate logistic regression analyzed the link between ApoE polymorphisms and CAD risk in populations at middle and high altitudes.

**Results:**

The data revealed significant differences in *APOE* gene ε3ε4 and ε4ε4 genotypes, as well as ε4 allele frequencies, 1256 CAD and non-CAD cases (*p* < 0.05). CAD patients with the ε4 allele had higher Apo-B/Apo-A1, Apo-B, and LDL-C levels than those with the ε2 or ε3 alleles. Furthermore, multifactorial logistic regression analysis indicated that the *APOE* gene’s ε3ε4 genotype (OR = 1.514, 95% CI = 1.087 - 2.109, *p* = 0.014) is an independent predictor for CAD.

**Discussion:**

These findings validated that the *APOE* gene’s ε3ε4 genotype is a potential predictor for CAD onset in Han Chinese individuals at middle and high altitudes.

## 1 Introduction

Coronary artery disease (CAD) is a chronic inflammatory disease caused by a combination of environmental and genetic factors. Furthermore, its development has been significantly associated with lipid metabolism regulation and the extent of coronary atherosclerosis. CAD has become a significant challenge for global cardiovascular health due to its complexity, long disease courses, and poor prognosis. Despite the rapid advancements in therapeutic techniques, CAD remains a major cause of death and morbidity globally (1, 2). China is home to an estimated 60–80 million permanent residents living at altitudes ≥ 1,500 m above sea level. The Qinghai–Tibet Plateau is a vast high-altitude region characterized by sustained hypobaric hypoxia, low ambient temperature, and low humidity. These extreme environmental stressors elicit pronounced systemic adaptations, with the cardiovascular system being preferentially affected, culminating in a distinct spectrum of pathophysiological changes (3, 4).Under chronic hypobaric hypoxia at moderate-to-high altitude, the right ventricle is subjected to sustained afterload, leading to compensatory right ventricular hypertrophy. To match the elevated myocardial oxygen demand, the coronary arteries undergo adaptive dilatation, thereby augmenting myocardial blood supply (5).These altitude-related pathophysiological changes substantially impair quality of life among permanent residents of moderate-to-high altitude regions, underscoring the urgent need to delineate the risk-factor profile of coronary artery disease (CAD) in this population.

**Figure 1:**
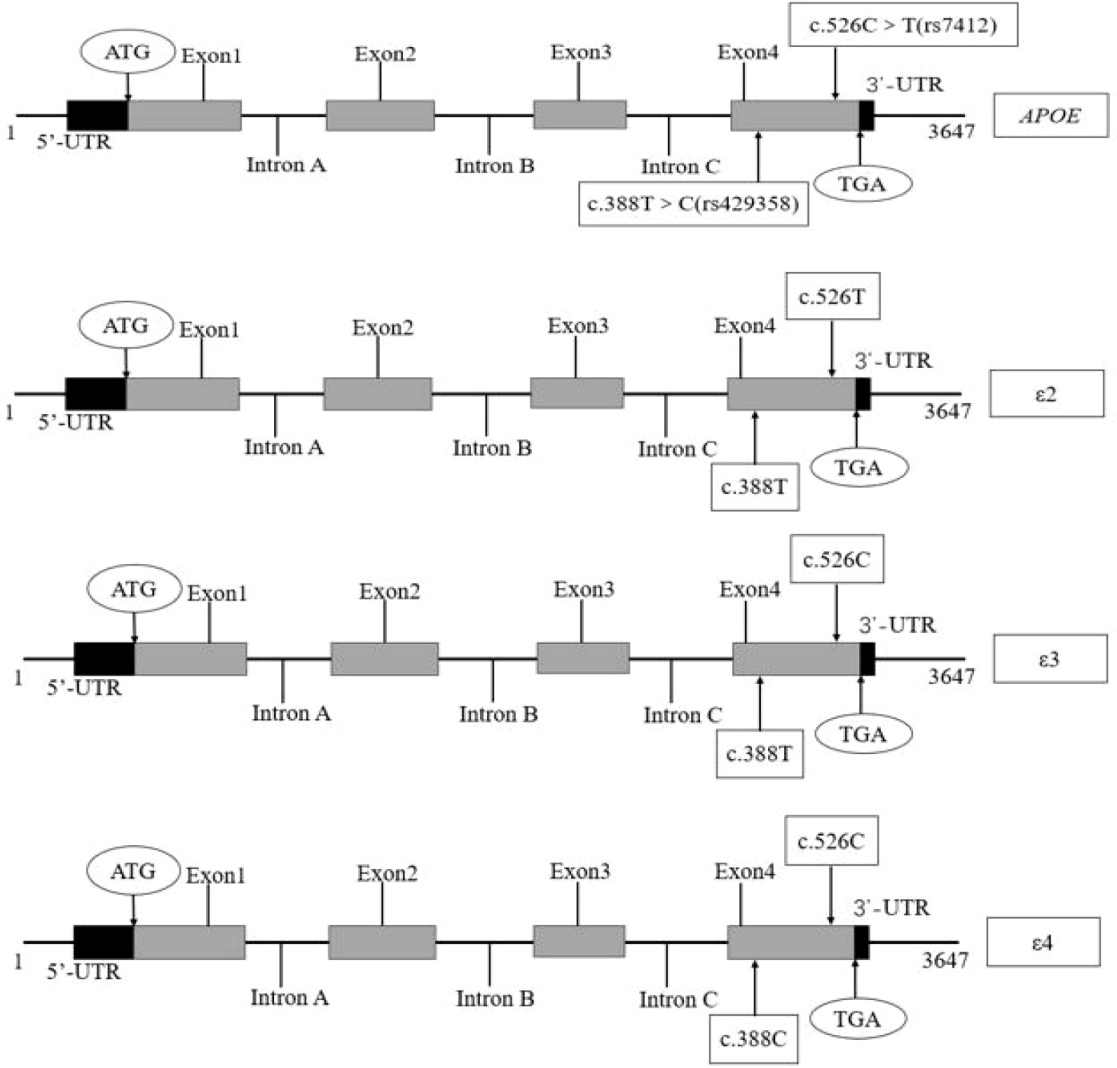
Schematic diagram of the gene structure and mutation sites of *APOE.*

**Figure 2:**
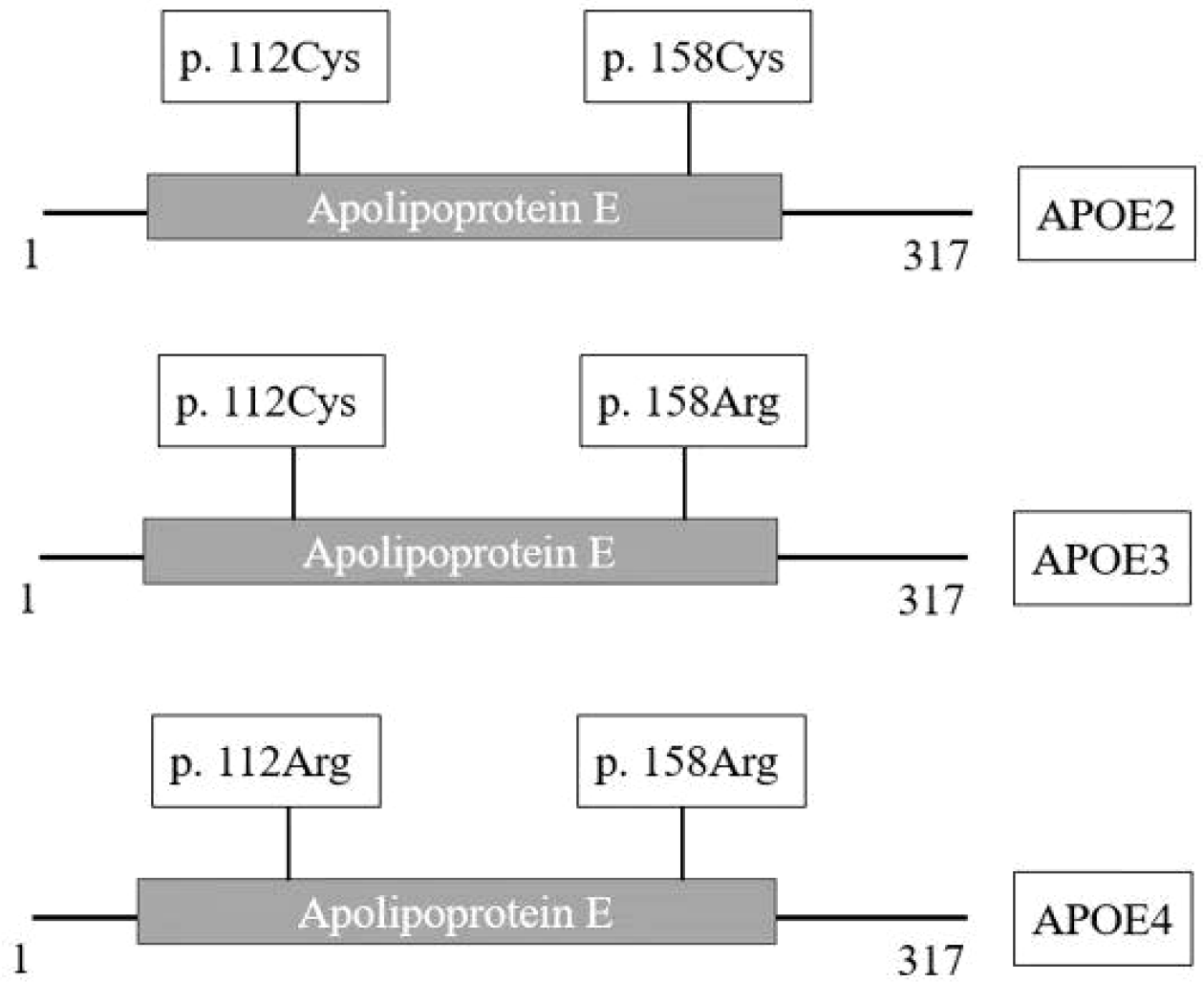
Schematic representation of the amino acid sites of the three APOE protein isoforms.

Previous studies have indicated that CAD is predominantly affected by aberrant regulation of lipid metabolism, which is mainly modulated by plasma lipoproteins. Apolipoprotein (APO) is an important component of plasma lipoproteins, as it serves as a ligand that binds lipoproteins to the receptor that determines the level of plasma lipoproteins. Therefore, APO gene mutations can affect lipid metabolism. Genetic susceptibility is the independent predictor that accounts for about half of CAD cases (6, 7). Thus, investigating the correlation between APO polymorphisms and the risk of developing coronary atherosclerosis is essential. The dyslipidemic metabolic regulation can initiate the atherosclerotic process, which, in turn, affects the onset and progression of CAD (8). The *APOE* gene, located on chromosome 19, is strongly associated with lipid metabolism (9). Moreover, this gene is polymorphically distributed and has two polymorphism loci, c. 388T > C (rs429358) and c. 526C > T (rs7412), resulting in three different alleles, ϵ2 (388T - 526T), ϵ3 (388T - 526C), and ϵ4 (388C - 526C) (**Table 1**; **Figure 1**). The most common type is ϵ3, referred to as the wild type, while the other two types are called mutants. Previous studies have identified 6 different genotypes, including three homozygotes and three heterozygotes (10, 11). The *APOE* gene encodes the APOE protein, which comprises 317 amino acids. Two sites have been observed at 112 (Cys to Arg) and 158 (Arg to Cys) amino acid positions. Moreover, APOE3, APOE2, and APOE4 all contain Cys, while APOE4 contains Arg at both these positions (**Table 1**; **Figure 2**).

**Table 1:** The two nucleotide mutation sites of the *APOE* genotype and the two amino acid mutation positions of the APOE apolipoprotein control.

Genotypes	Variant nucleotide position		*APOE* subtype	Amino acid position (112)	Amino acid position (158)
	c.388 (rs429328)	c.526 (rs7412)			
ϵ2/ϵ2	TT/TT		*APOE*2	Cys	Cys
ϵ2/ϵ3	TT/TC				
ϵ3/ϵ3	TT/CC		*APOE*3	Cys	Arg
ϵ2/ϵ4	TC/TC				
ϵ3/ϵ4	TC/CC		*APOE*4	Arg	Arg
ϵ4/ϵ4	CC/CC				

Cys, Cysteine; Arg, arginine.

The *APOE* gene modulates lipid metabolism and has multiple alleles, with ϵ2, ϵ3, and ϵ4 being the most widely studied. Research has shown that the ϵ4 allele and genotypes ϵ3ϵ4 and ϵ4ϵ4 are associated with a greater incidence of CAD in low-altitude regions (12). However, the ϵ2 allele and genotypes ϵ2ϵ2 and ϵ2ϵ3 do not significantly increase CAD risk (13). Similarly, lipid-lowering drugs are often less effective in reducing LDL-C levels in ϵ4 carriers. The distribution of *APOE* allele frequencies shows significant heterogeneity among various ethnic groups and geographical regions (14). Genetic susceptibility is a significant risk factor, contributing to approximately half of CAD cases. Therefore, it is crucial to investigate the relationship between *APOE* polymorphisms and the risk of developing coronary atherosclerosis. The influence of APOE polymorphisms on CAD is further complicated by their interactions with a range of genetic and environmental factors. In China, there is significant regional variation in the distribution of *APOE* alleles. CAD presents unique challenges at middle and high altitudes due to the physiological stresses inherent to these environments. The relationship between *APOE* gene polymorphisms and CAD risk in China’s middle and high-altitude regions remains inadequately studied.

Therefore, this study aims to elucidate the role of *ApoE* genotypes in CAD risk and examine the interrelationships between lipid profiles and *ApoE* alleles and genotypes within the Han Chinese population residing at high altitudes to determine the association between *APOE* gene polymorphisms and CAD risk in China’s middle and high-altitude regions. Furthermore, our findings delineate evidence-based, genotype-guided lifestyle and pharmacological interventions that can be implemented during the pre-symptomatic phase to curtail incident angina and myocardial infarction and, ultimately, reducing the mortality rate associated with CAD.

## 2 Methods

### 2.1 Research participants

This prospective study included 1256 participants from the Qinghai Provincial Specialized Hospital for Cardiovascular Diseases between March 2023 and December 2023. Of these, 628 (M: F = 440: 188) were included in the CAD experimental cohort, while 628 (M: F = 330: 298) were included in the non-CAD control cohort. All participants were Han Chinese individuals who had resided in China for an extended period at middle and high altitudes (as defined by the International Mountain Society: middle altitude is 1500–2500 m, and high altitude is ≥ 2500 m). Furthermore, the selected participants had no history of intermarriage. The CAD group primarily comprised patients with at least a 50% stenosis of the main coronary artery or major branches, or who were hospitalized for CAD. The control group mainly comprised individuals without cardiovascular disease. Diabetes mellitus (DM) patients were defined as individuals who, at the time of hospital admission, had a fasting or anytime blood glucose level of ≥ 7.0 mmol/L or ≥ 11.1 mmol/L, respectively, or a 2-hour postprandial blood glucose level of ≥ 11.1 mmol/L. Hypertension was defined as individuals with a mean systolic or diastolic blood pressure of at least 140 and 90 mmHg, respectively. This study was approved by the Ethics Committee of Qinghai Provincial Cardiovascular and Cerebrovascular Disease Specialist Hospital. Informed consent was acquired from each patient before their participation.

### 2.2 Data collection

Each participant’s general data was collected, including age, sex, long-term residence altitude, smoking history, and alcohol consumption history. Moreover, fasting venous blood was collected from each participant early morning to assess the levels of total cholesterol (TC), apolipoprotein B (Apo-B), triglycerides (TG), LDL-C, apolipoprotein A1 (Apo-A1), high-density lipoprotein cholesterol (HDL-C), the ratio of Apo-B/Apo-A1, and lipoprotein a [Lp(a)]. Further, before measuring systolic and diastolic blood pressure with a sphygmomanometer, the subjects were instructed to remain fully at rest for more than 5 minutes. Each patient’s average systolic and diastolic blood pressure values were included in the study.

### 2.3 DNA extraction and gene typing

Venous blood (approximately 2 mL) was collected from each subject. The TIA Namp Blood DNA kit DP 318 (Tiangen Biochemical Technology Co., Beijing, China) was employed for the genomic DNA extraction, which was then stored at -25 ± 5 °C, protected from light, per the instructions provided in the kit. The *APOE* gene polymorphism typing was conducted using a PCR-fluorescent probe detection kit (Youzhiyou Medical Technology Co., Ltd., Wuhan, Hubei, China). PCR was performed according to the kit’s protocol: UNG treatment at 37 °C for 10 minutes, a pre-denaturation at 95 °C for 5 minutes, and then 40 thermal cycles (15 s at 95 °C and 45 s at 60 °C). After PCR amplification, the products were hybridized to GeneChip probes to determine the sample’s genotypes.

### 2.4 Statistical analysis

SPSS 26.0 was employed for all the statistical analyses. The categorical variables data are presented as numbers and percentages, while the continuous variables data are presented as mean ± standard deviation. The intergroup differences in the categorical variables data were analyzed using the Fisher test, while the continuous variables data were analyzed using a Student *t-test*. Furthermore, genotype ratios, allele frequencies, and Hardy-Weinberg equilibrium (HWE) in the CAD and control groups were assessed using a Chi-square test. Moreover, logistic regression was used to elucidate the association between the *APOE* gene polymorphism and CAD. The results were deemed statistically significant at *p* < 0.05.

## 3 Results

### 3.1 Characteristics of the subjects

**Table 2** presents the demographic and clinical characteristics of the entire study cohort. The CAD group comprised 440 (70.06%) males and 188 (29.94%) females, while the control group had 330 males (52.55%) and 298 females (47.45%).

**Table 2:** Clinical characteristics of patients and subjects with CAD.

	CAD patients (n = 628)	Controls (n = 628)	χ2	*P*-values
Gender				
Female, n (%)	188 (29.94%)	298 (47.45%)	40.611	<0.001
Male, n (%)	440 (70.06%)	330 (52.55%)		
Age, years				
< 60, n (%)	254 (40.45%)	253 (40.29%)	0.003	0.954
≥ 60, n (%)	374 (59.55%)	375 (59.71%)		
Altitudes				
1500–2500 m, n (%)	397 (63.22%)	371 (59.08%)	2.265	0.132
≥ 2500 m, n (%)	231 (36.78%)	257 (40.92%)		
History of smoking, n (%)	224 (35.67%)	128 (20.38%)	36.377	<0.001
History of drinking, n (%)	103 (16.40%)	99 (15.76%)	0.094	0.759
Hypertension, n (%)	336 (53.50%)	294 (46.82%)	5.618	0.018
Diabetes, n (%)	162 (25.80%)	107 (17.04%)	14.301	<0.001
			*t* values	*P* values
TC (mmol/L)	3.920 ± 1.599	4.068 ± 1.075	-1.916	0.750
TG (mmol/L)	1.840 ± 1.170	1.785 ± 1.346	0.771	0.527
HDL-C (mmol/L)	1.069 ± 0.240	1.146 ± 0.287	-5.129	<0.001
LDL-C (mmol/L)	2.201 ± 0.809	2.322 ± 0.851	-2.571	0.916
Apo-A1 (g/L)	1.107 ± 0.189	1.149 ± 0.229	-3.525	<0.001
Apo-B (g/L)	0.735 ± 0.227	0.753 ± 0.240	-1.324	0.956
Apo-B/Apo-A1	0.679 ± 0.229	0.672 ± 0.232	0.545	0.487
Lp(a) (mmol/L)	292.206 ± 271.117	243.337 ± 239.656	3.384	0.004

P values < 0.001, significant difference

In the CAD group, 254 (40.45%) individuals were < 60 years old, while 374 (59.55%) were ≥ 60 years old. A total of 397 (63.22%) cases indicated long-term survival at middle altitude, of which 224 (35.67%) and 103 (16.40%) had a history of smoking and alcohol consumption, while 336 (53.50%) and 162 (25.80%) had hypertension and DM, respectively. The proportion of these variables was lower in the control than in the CAD group. Furthermore, the two groups differed significantly in the number of males (*p*<0.001), smoking history (*p*<0.001), hypertension (*p =* 0.018), and DM (*p*<0.001). The levels of TG, Apo-B/Apo-A1, Lp(a), and age in the CAD group were higher than those in the control group. Other variables, including altitude, TC, LDL-C, HDL-C, Apo-B, and Apo-A1, showed higher levels in the control population than in the CAD group. Significant differences were observed in age (*p*<0.001), HDL-C (*p*<0.001), Apo-A1 (*p*<0.001), and Lp(a) (*p =* 0.004< 0.05).

### 3.2 Distribution of *APOE* genotypes and alleles in the research population

**Table 3** shows that the distribution of *APOE* genotypes was in balance with the expected values under Hardy-Weinberg equilibrium in the CAD (χ^2^ = 2.397, *p =* 0.663) and control (χ^2^ = 3.944, *p =* 0.414) groups. Furthermore, the percentage of ϵ3ϵ3 was higher in the control group (71.49%) than in the CAD group (67.04%). This was followed by ϵ3ϵ4, which had a higher percentage in the CAD group (18.15% vs 11.94% in the control group). Moreover, the ϵ2ϵ3 and ϵ2ϵ4 percentages of the control group (13.38% and 2.07%, respectively) were higher than those of the CAD group (11.47% and 1.43%, respectively). The ϵ4ϵ4 percentage of the CAD group was greater than that of the control group (1.75% vs 0.48%), while the ϵ2ϵ2 percentage of the CAD group was lower than that of the control group (0.16% vs 0.64%). **Table 3** shows that the difference in *APOE* genotype distribution between these two groups was statistically significant (χ2 = 16.783, *p =* 0.004< 0.05), as was the difference in allele distribution (χ^2^ = 13.893, *p =* 0.001 < 0.05).

**Table 3:** Distribution of *APOE* genotypes and alleles frequencies in the CAD and control group.

Genotypes, n (%)	CAD patients (628)	Controls patients (628)	χ2	*P*-values	χ2	*P*-values
ϵ2/ϵ2	1 (0.16%)	4 (0.64%)	1.807	0.179	16.783	0.004
ϵ2/ϵ3	72 (11.47%)	84 (13.38%)	1.451	0.228		
ϵ2/ϵ4	9 (1.43%)	13 (2.07%)	0.740	0.390		
ϵ3/ϵ3	421 (67.04%)	449 (71.49%)	2.932	0.087		
ϵ3/ϵ4	114 (18.15%)	75 (11.94%)	9.473	0.002		
ϵ4/ϵ4	11 (1.75%)	3 (0.48%)	4.623	0.032		
HWE (χ2, p)	χ2 = 2.397, P = 0.663	χ2 = 3.944, p = 0.414				
Alleles, n (%)						
ϵ2	83 (6.61%)	105 (8.36%)	2.783	0.095	13.895	0.001
ϵ3	1028 (81.85%)	1057 (84.16%)	2.373	0.123		
ϵ4	145 (11.54%)	94 (7.48%)	12.027	0.001		

HWE, Hardy Weinberg Equilibrium.

### 3.3 Distribution patterns of *APOE* genotypes and alleles among CAD

This study analyzed the clinical characteristics, laboratory indices, and *APOE* genotypes of CAD patients in the middle and high altitudes of the Han population (**Table 4**). In the CAD group, HDL-C levels (the unit is mmol/L) were significantly higher in the ϵ2ϵ3 genotype (1.088 ± 0.245) than in ϵ3ϵ3 (1.083 ± 0.245) and ϵ3ϵ4 (1.022 ± 0.214 mmol/L) genotypes (*p =* 0.046). LDL-C levels in CAD patients with ϵ3ϵ4 genotype (2.372 ± 0.892) were significantly greater than those with ϵ3ϵ3 (2.219 ± 0.789), and ϵ2ϵ3 (1.842 ± 0.704) (*p*<0.001). Furthermore, the Apo-B/Apo-A1 levels in ϵ3ϵ4 (0.747 ± 0.228) genotype patients were significantly higher than in the ϵ2ϵ3 (0.581 ± 0.199) and ϵ3ϵ3 (0.675 ± 0.225) genotype patients (*p*<0.001). The HDL-C level (the unit is mmol/L) of the ϵ2 allele in CAD (1.087 ± 0.243) was significantly higher than that of ϵ3 (1.083 ± 0.245) and ϵ4 (1.021 ± 0.216) (*p =* 0.034 < 0.05). Moreover, the LDL-C level (the unit is mmol/L) of the ϵ4 allele (2.384 ± 0.882) was significantly higher than that of the ϵ3 (2.219 ± 0.789) and ϵ2 (1.833 ± 0.703) (*p*<0.001). Further, ϵ4 had a higher level of Apo-B (0.797 ± 0.241) than ϵ3 (0.735 ± 0.224) and ϵ2 (0.637 ± 0.200), and the difference between the three alleles (*p*<0.001) was statistically significant. The Apo-B/Apo-A1 level of ϵ4 (0.754 ± 0.240) was significantly higher than that of ϵ3 (0.675 ± 0.225), ϵ2 (0.578 ± 0.199) (*p*<0.001).

**Table 4:** Distributional characteristics of *APOE* in Han Chinese CAD population in middle and high-altitude areas.

	ϵ2/ϵ3 (72)	ϵ3/ϵ3 (421)	ϵ3/ϵ4 (114)	χ2	*P*-values	ϵ2 (82)	ϵ3(607)	ϵ4(134)	χ2	*P*-values
Gender										
Males, n (%)	49	290	84	1.076	0.585	58	423	100	1.257	0.529
Females, n (%)	23	131	30			24	184	34		
Age, years										
< 60, n (%)	30	168	47	0.147	0.933	34	245	56	0.136	0.937
≥ 60, n (%)	42	253	67			48	362	78		
Altitudes										
1500–2500 m, n (%)	46	257	83	5.422	0.066	51	386	93	1.812	0.404
≥ 2500 m, n (%)	26	164	31			31	221	41		
History of smoking, n (%)	21	149	44	1.712	0.429	24	214	54	2.73	0.259
	51	272	70			58	393	80		
History of drinking, n (%)	9	65	24	2.779	0.239	11	98	28	2.397	0.298
	63	356	90			71	509	106		
hypertension, n (%)	41	230	54	2.275	0.32	45	325	64	1.64	0.444
	31	191	60			37	282	70		
Diabetes, n (%)	20	108	28	0.273	0.886	21	156	34	0.015	0.997
	52	313	86			61	451	100		
TC (mmol/L)	3.574 ± 0.861	3.976 ± 1.824	3.971 ± 1.051		0.142	3.560 ± 0.862	3.976 ± 1.824	3.987 ± 1.046		0.113
TG (mmol/L)	1.838 ± 1.007	1.778 ± 1.100	2.001 ± 1.187		0.16	1.831 ± 1.001	1.778 ± 1.100	2.043 ± 1.461		0.085
HDL-C (mmol/L)	1.088 ± 0.245	1.083 ± 0.245	1.022 ± 0.214		0.046	1.087 ± 0.243	1.083 ± 0.245	1.021 ± 0.216		0.034
LDL-C (mmol/L)	1.842 ± 0.704	2.219 ± 0.789	2.372 ± 0.892		<0.001	1.833 ± 0.703	2.219 ± 0.789	2.384 ± 0.882		<0.001
Apo-A1 (g/L)	1.126 ± 0.196	1.114 ± 0.191	1.076 ± 0.170		0.113	1.125 ± 0.194	1.114 ± 0.191	2.384 ± 0.882		0.1
Apo-B (g/L)	0.640 ± 0.199	0.735 ± 0.224	0.792 ± 0.242		<0.001	0.637 ± 0.200	0.735 ± 0.224	0.797 ± 0.241		<0.001
Apo-B/Apo-A1	0.581 ± 0.199	0.675 ± 0.225	0.747 ± 0.228		<0.001	0.578 ± 0.199	0.675 ± 0.225	0.754 ± 0.240		<0.001
Lp(a) (mmol/L)	271.596 ± 270.840	301.443 ± 268.067	285.322 ± 288.0337		0.634	268.681 ± 270.103	301.443 ± 268.067	279.954 ± 287.393		0.529

### 3.4 Investigation of the correlation between *APOE* gene polymorphism and CAD

The univariate logistic regression analysis revealed that gender, smoking history, hypertension, and diabetes were the independent predictor for CAD. Corrected OR and *p-*values were obtained using multifactor logistic regression. For CAD, the independent predictors in the Han Chinese population at middle and high altitudes (*p* < 0.05) were gender, smoking history, hypertension, and DM. Because the ϵ2 and ϵ4 alleles exert diametrically opposed effects on lipid metabolism, ϵ2ϵ4 carriers were excluded from the APOE–CAD association analysis (15). **Table 5** indicates that ϵ3ϵ4 is an independent predictor for CAD in the Han Chinese population at middle- and high-altitude locations. The corrected *p-*value and OR of ϵ3ϵ4 were also assessed (OR = 1.514, 95% CI = 1.087 - 2.019, *p =* 0.014< 0.05).

**Table 5:** Multifactorial logistic regression analysis of factors associated with CAD.

Variables	Genotypes	Univariate OR (95% *CI*)	*P-*values	Multivariate OR (95% *CI*)	*P-*values
Gender (Male/Female)		2.113 (1.676-2.665)	<0.001	2.006 (1.573-2.557)	<0.001
Age (< 60/≥ 60)		0.993 (0.793-1.245)	0.954	0.989 (0.786-1.243)	0.922
Altitudes (< 2500/≥ 2500)		0.840 (0.669-1.504)	0.132	0.867 (0.685-1.099)	0.238
Smoker (yes/no)		2.166 (1.681-2.791)	<0.001	2.095 (1.619-2.712)	<0.001
Alcoholism (yes/no)		1.048 (0.776-1.417)	0.759	1.029 (0.759-1.395)	0.853
hypertension, n (%)		1.307(1.047-1.632)	0.018	1.308(1.046-1.637)	0.019
Diabetes, n (%)		1.693 (1.287-2.227)	<0.001	1.676 (1.271-2.211)	<0.001
Genotypes of *APOE*					
	ϵ3/ϵ3	1.000 (reference)	——	1.000 (reference)	——
	ϵ2/ϵ2	0.267 (0.030-2.395)	0.238	0.345 (0.037-3.217)	0.350
	ϵ2/ϵ3	0.914 (0.650-1.287)	0.607	0.925 (0.648-1.320)	0.667
	ϵ3/ϵ4	1.621 (1.177-2.233)	0.003	1.514 (1.087-2.109)	0.014
	ϵ4/ϵ4	3.911 (1.083-14.114)	0.037	2.726 (0.737-10.086)	0.133

P-values < 0.001, significant difference

## 4 Discussion

Multiple factors are associated with the development of CAD, specifically environment, lifestyle, and genetic predisposition (16). CAD development is associated with dyslipidemia levels in the body; therefore, it is a risk factor for CAD (17, 18). APOE, a 34-kDa apolipoprotein with isoform-dependent structure and function, serves as the principal ligand for receptor-mediated clearance of LDL-C, VLDL-C, HDL-C and chylomicrons. Rajesh Chaudhary et al. demonstrated that ϵ2 carriers exhibit elevated triglycerides and reduced total cholesterol, whereas ϵ4 carriers display higher total and LDL-cholesterol concentrations (19). *APOE* gene polymorphism has a significant effect on fatty acid transport, mobilization ability, mitochondrial oxidation efficiency and metabolic flexibility in hypoxic middle and high altitude areas. When oxygen-dependent carbohydrate oxidation becomes less favorable, genes affect an individual ‘s ability to maintain lipid-based energy production (20). APOE predisposes to coronary artery disease through three converging pathways: allele-specific modulation of cholesterol metabolism, propagation of chronic low-grade inflammation, and amplification of vascular oxidative stress. Under physiological conditions, the endothelium maintains vascular tone and patency. Dysregulated cholesterol metabolism disrupts this homeostasis: oxidized LDL accumulates in the intima, elicits endothelial dysfunction, and recruits innate immune cells that amplify local and systemic inflammation. Shifts the hemostatic balance toward a pro-thrombotic phenotype, and accelerates atherosclerotic plaque progression—collectively increasing CAD risk (21).It has been revealed that the association of the *APOE* gene with CAD, however, the distribution of *APOE* alleles and their genotypes, vary across regions and races (22). For example, Europe has a high and Asia has a low ϵ4 allele frequency (14). However, data on *APOE* gene distribution in the Chinese population are limited. This study is the first to characterize *APOE* gene frequency and clinical features in a Han Chinese population residing in the middle- and high-altitude regions of China.

This research investigated the *APOE* gene polymorphisms and their allele frequencies in the CAD population residing at middle and high altitudes in China. The results (**Table 3**) revealed that, overall, the allele frequencies were ϵ3 > ϵ4 > ϵ2 and ϵ4 > ϵ3 > ϵ2 in the CAD and control populations, respectively. This distribution is consistent with the results of Gerdes LU et al, indicating that in Asian countries, the most common allele is ϵ3, while ϵ2 and ϵ4 are relatively rare (23). The frequencies of the *APOE* allele in the control group differed between the population living at middle and high altitudes or in the Chinese plains (24, 25), indicating that locations at the middle and high altitudes influence the frequencies of the *APOE* allele. The elevated APOE ϵ4 frequency in cold, high-altitude regions may reflect climatic selection: low temperatures curtail outdoor activity and ultraviolet-B exposure, reduce cutaneous vitamin D synthesis, and thereby favor ϵ4, which sustains higher 25-hydroxyvitamin D levels under hypovesic environments (26). Moreover, this study revealed that TG, Apo-B/Apo-A1, Lp(a), and altitude levels were elevated in the CAD group relative to the control group. Abnormal lipid levels, are a significant risk factor for CAD. *APOE* gene polymorphisms are associated with lipid levels (27). Relative to the wild-type ϵ3 isoform, ϵ2 exhibits markedly reduced binding affinity for the low-density lipoprotein receptor (LDLR), impairing the hepatic uptake of chylomicron and VLDL remnants and promoting their prolonged circulation. Conversely, ϵ4 displays greater LDLR affinity, which facilitates accelerated hepatic clearance of triglyceride-rich lipoproteins and ultimately raises plasma total and LDL-cholesterol concentrations. It was revealed that the allele with the strongest affinity for LDL-C was ϵ4, followed by ϵ3 and ϵ2. Here, APOE ϵ4 allele carriers showed higher levels of TG, LDL-C, TC, HDL-C, Apo-B, Apo-A1, Apo-B/Apo-A1, and Lp(a) than those carrying the ϵ2 or ϵ3 alleles. Furthermore, the observed differences were statistically significant. APOE ϵ4 allele carriers showed the highest levels of TG, Apo-B, LDL-C, and Apo-B/Apo-A1, while indicating the lowest levels of HDL-C and Apo-A1. Moreover, compared to the ϵ2 and ϵ3 alleles, the ϵ4 allele increased the risk of CAD, consistent with the conclusions drawn by Luo JQ et al. (12). Consistently, carriage of the APOE ϵ4 allele has been associated with both an increased risk of coronary heart disease and more severe angiographic stenosis; furthermore, ϵ4-positive patients with acute coronary syndrome exhibit a significantly higher rate of major adverse cardiovascular events (MACE) during long-term follow-up (28). Statistical analysis revealed significant differences in HDL-C, LDL-C, Apo-B, and Apo-B/Apo-A1 levels among the three *APOE* genotypes, ϵ2ϵ3, ϵ3ϵ3, and ϵ3ϵ4 (*p* < 0.05). Furthermore, the ϵ4 allele was observed to be an independent predictor, while the ϵ2 allele served as a protective factor for CAD, consistent with previous studies. The comparison of ϵ2 carriers with ϵ3 and ϵ4 carriers revealed that the former population had a 20% reduced risk of CAD (29–31). Since the prevalence of the purebred ϵ3ϵ3 type (67.04%) in this study population, the ϵ3ϵ3 genotype was employed for comparative analysis (32). This study identified the ϵ3ϵ4 *APOE* genotype as a potential independent predictor for CAD development in Han Chinese individuals residing at middle and high altitudes, this is the first study to delineate the association between *APOE* gene polymorphisms and coronary artery disease among Han Chinese residing permanently at middle and high altitudes. The multifactorial logistic regression analysis validated this association. The *APOE* ϵ2 allele reduces the risk of CAD by upregulating the LDL-C receptor, thus altering lipid levels and promoting atherosclerosis (33). Compared with ϵ2 and ϵ3 carriers, ϵ4 carriers have been observed to elevate plasma lipoprotein levels and promote atherosclerosis susceptibility. This, in turn, increases the risk of CAD among Han Chinese residing at middle and high altitudes (34).

It was concluded that the risk of CAD in the ϵ3ϵ4 and ϵ4ϵ4 populations was 1.6 and 3.9 times higher than in the ϵ3ϵ3 population, respectively. Whereas the risk of CAD in ϵ2ϵ2 and ϵ2ϵ3 populations was 0.2 and 0.6 times higher than in the ϵ3ϵ3 population, respectively. Therefore, it was proposed that ϵ3ϵ4 and ϵ4ϵ4 genotypes are independent predictors for CAD, whereas ϵ2ϵ2 and ϵ2ϵ3 genotypes are protective factors for CAD among Han Chinese residing at middle and high altitudes. This conclusion is consistent with that of previous studies (34). After adjusting for several common CAD independent predictors, only ϵ3ϵ4 was found as an independent predictor for CAD in the concerned population. In the present study, the ϵ4ϵ4 genotype was excluded as an independent predictor for CAD, most likely because of its low prevalence and the consequently limited statistical power. Larger, multicohort studies are warranted to definitively clarify the role of ϵ4ϵ4 in high-altitude Han Chinese populations. Related UK studies have reported that the hazard ratios for the ϵ2ϵ3 and ϵ3ϵ4 genotypes compared to the ϵ3ϵ3 population were (0.85 - 1.10) and (0.97 - 1.15), respectively (35). Furthermore, the population in the plains of southern China also indicated that the ϵ3ϵ4 genotype is an independent predictor and ϵ2ϵ3 is a protective factor for CAD (15), consistent with the results of this study. This study also included the altitude at which the patients were located and the data revealed no significant correlation between altitude and the risk of developing CAD (OR = 0.867, 95% CI = 0.685 - 1.099, *p =* 0.238), which was not consistent with the study of Al-Huthi MA et al., who revealed that high altitude is an independent predictor for CAD (36). These disparate findings may partly reflect dietary heterogeneity: the high-altitude cohort consumed a diet richer in cardioprotective micronutrients (e.g., ω-3 polyunsaturated fatty acids, polyphenols and dietary fiber), which could counterbalance the pro-atherogenic effects of the ϵ4 allele. The analysis of the specific mechanism of the *APOE* gene in the concerned population revealed that serum LDL-C levels were elevated in most atherosclerosis patients. Furthermore, a study conducted in Ecuador demonstrated a potential protective effect of middle and high altitudes on the risk of ischemic heart disease (37). Therefore, additional data are required to further investigate the correlation between altitude and the risk of CAD development. This study found that the ϵ3ϵ4 genotype was an independent predictor for CAD development in the Han Chinese population at middle and high altitudes. Furthermore, this study provides new evidence for individuals with the ϵ3ϵ4 genotype and recommends improving their lifestyle through regular aerobic exercise, a low-salt, low-fat diet, early assessment for CAD, and the judicious use of medications for secondary prevention of CAD. There are some limitations in this study. First, its retrospective design and single-center setting inevitably constrained sample size and data completeness, introducing potential information and selection biases. Notably, although all participants resided permanently at moderate-to-high altitude, we lacked serial recordings of oxygen saturation and hemoglobin levels, precluding appraisal of hypoxemic burden. Second, coronary artery disease is a polygenic and multifactorial disorder; however, medication histories could not be independently verified. Consequently, we were unable to adjust for prior use of statins or other lipid-lowering agents, which may have attenuated the observed genotype–phenotype associations and hampered evaluation of gene–environment interactions.

## 5 Conclusions

In summary, it was revealed that in the Chinese Han CAD population living at middle and high altitudes, the *APOE* ϵ3ϵ3 genotype had the highest frequency, while the ϵ2ϵ2 genotype had the lowest. Furthermore, the multifactorial logistic regression analyses revealed that gender, smoking, hypertension, DM, and *APOE* ϵ3ϵ4 genotype are the independent predictor for CAD. This study provided a theoretical basis for early risk assessment of CAD in individuals with the ϵ3ϵ4 genotype. These findings can help prevent CAD and formulate diagnostic strategies for high-risk CAD individuals living in the middle and high altitudes of China.

## Data Availability

The raw data supporting the conclusions of this article will be made available by the authors, without undue reservation.
